# Tackling the Burden of Osteoarthritis as a Health Care Opportunity in Indigenous Communities—A Call to Action

**DOI:** 10.3390/jcm9082393

**Published:** 2020-07-27

**Authors:** Penny O’Brien, Samantha Bunzli, Ivan Lin, Tilini Gunatillake, Dawn Bessarab, Juli Coffin, Gail Garvey, Michelle Dowsey, Peter Choong

**Affiliations:** 1 Department of Surgery, St Vincent’s Hospital Melbourne, The University of Melbourne, Melbourne, VIC 3000, Australia; samantha.bunzli@unimelb.edu.au (S.B.); gunatillake.t@unimelb.edu.au (T.G.); mmdowsey@unimelb.edu.au (M.D.); pchoong@unimelb.edu.au (P.C.); 2 Western Australian Centre for Rural Health, The University of Western Australia, Geraldton, WA 6530, Australia; ivan.lin@uwa.edu.au; 3 Centre for Aboriginal Medical and Dental Health, The University of Western Australia, Perth, WA 6000, Australia; dawn.bessarab@uwa.edu.au; 4 Telethon Kids Institute, Broome, WA 6725, Australia; juli.coffin@telethonkids.org.au; 5 Inclusive Health, St Vincent’s Health Australia, Melbourne, VIC 3000, Australia; gail.garvey@svha.org.au; 6 Menzies School of Health Research, Brisbane, QLD 4000, Australia

**Keywords:** osteoarthritis, Indigenous health, chronic disease

## Abstract

Osteoarthritis is a highly prevalent and disabling disease, causing a significant individual and socioeconomic burden worldwide. Until now, there has been a dearth of research exploring the impact of osteoarthritis in global Indigenous communities. Osteoarthritis has a similar risk factor profile to many chronic diseases that disproportionately affect Indigenous peoples. In this editorial, we argue that osteoarthritis and associated mobility restrictions play a central role in the chronic disease profile of Indigenous peoples. We present a call to action for clinicians and health care providers, researchers and policymakers to begin to recognise the interrelated nature of osteoarthritis and chronic disease. We have an opportunity to change the way we do business, to improve access to culturally secure osteoarthritis care and the health and wellbeing of Indigenous communities.

## 1. Why Is Osteoarthritis Important?

Osteoarthritis is the single most common cause of disability in older adults, affecting upwards of 20% of the adult population [1,2,3]. As the largest contributor to the global burden of musculoskeletal disorders, osteoarthritis and its attendant disabilities cause commensurate individual and socioeconomic costs worldwide [1,2]. Sociodemographic trends such as population ageing and increased prevalence of modifiable risk factors such as obesity and sedentary lifestyle are driving an increase in the prevalence of osteoarthritis, such that the burden of disease is growing more rapidly than any other health condition [2]. The number of people affected by osteoarthritis is projected to increase by 58% over the next two decades, the sharpest upward trajectory across all musculoskeletal conditions [4]. Experts in the field have drawn attention toward the growing, ubiquitous health and socioeconomic burden of osteoarthritis [2], yet until now there has been little consideration of how osteoarthritis impacts Indigenous populations.

Indigenous populations in Australia (Aboriginal and Torres Strait Islander), New Zealand (Māori), Canada (First Nations, Inuit and Métis) and the United States of America (Alaskan Native and American Indian) continue to face substantial economic, health and social disadvantage as a legacy of marginalization and colonial practices. Through the effects of colonization, Indigenous populations have experienced rapid increases in chronic conditions such as diabetes, obesity, cardiovascular disease and respiratory illnesses [5,6,7]. Globally, over 50% of Indigenous adults aged over 35 have at least one chronic disease [7]. In Australia, this proportion increases to approximately 90% for Indigenous Australians aged 55 and over [8]. High rates of risk factors such as obesity, smoking, lower physical activity levels and lower levels of educational attainment in Indigenous populations and the resultant poorer health outcomes are well documented. The proportion of Indigenous peoples who smoke in the United States of America (USA), Canada, Australia and New Zealand is reported between 23 and 59% [8,9,10], compared to 14–16% in the wider population [10,11,12]. Indigenous Australian adults are also 1.6 × more likely to be obese than non-Indigenous Australians [13]. Osteoarthritis has a similar risk factor profile and commonly coexists with chronic conditions that disproportionately affect Indigenous peoples. Symptoms such as pain, stiffness and impaired mobility mean osteoarthritis is a leading cause of physical activity limitation [14]. Reduced mobility impacts on participation in work, sport, family, daily function, emotional wellbeing, cultural participation and one’s ability to self-mange co-morbid chronic conditions. As a result, people with osteoarthritis die younger than those without osteoarthritis [15]. To date, there is a dearth of research acknowledging the interrelated nature of osteoarthritis and chronic health conditions in an Indigenous context. 

## 2. What Do We Know and What Are the Gaps?

Indigenous populations of Australia, New Zealand, Canada and the USA experience higher prevalence and greater burden of disease associated with osteoarthritis. Indigenous Australians experience rates of osteoarthritis between 1.2 and 1.5 higher than non-Aboriginal Australians [13], and the burden of disease is greater (31 disability-adjusted life years per 1000 people for Indigenous versus 22 per 1000 people for non-Indigenous Australians) [16]. Māori who undergo joint replacement surgery for osteoarthritis are younger and have worse preoperative function and postoperative functional improvements than non-Māori [17]. Canadian First Nations experience osteoarthritis prevalence twice that of non-First Nations [18], whilst American Indians have the highest prevalence of arthritis of any population group in the USA [19]. Despite this, Indigenous peoples’ access care for osteoarthritis at substantially lower rates than non-Indigenous people. In Australia, Indigenous peoples’ access primary care services [6] and total joint replacements at half the expected rate based on incidence [20]; similarly, First Nation Canadians also access orthopaedic outpatient consultations, specialist services (such as rheumatology and orthopaedics) and joint replacements at substantially lower rates than non-First Nations [18]. 

A systematic review of Indigenous populations in Australia, Canada, New Zealand and USA highlighted a lack of evidence in this area [21], while another Australian review concluded that the management of osteoarthritis in Indigenous Australians is an unmet health need [22]. The health needs of Indigenous peoples are complex, and until now, priority has been given to conditions that directly contribute to the disparities in life expectancy and the ‘’health gap’’, such as diabetes, cardiovascular disease and child and maternal health. However, as the leading cause of mobility restriction, osteoarthritis contributes indirectly to disparities in life expectancy and the health gap. The question therefore arises of how to raise awareness about the central role that osteoarthritis plays in the chronic disease story. 

## 3. Call to Action 

Chronic disease is a serious threat to the wellbeing of Indigenous communities worldwide. By improving the musculoskeletal health of Indigenous peoples, we have an opportunity to change the chronic disease landscape and wellbeing of Indigenous communities. We therefore call on clinicians and health care providers, researchers and policymakers to (see Table 1):

(1) Recognise that osteoarthritis is a leading cause of mobility restriction among Indigenous peoples and is therefore a central piece in the chronic disease puzzle. Building capacity in the Indigenous health workforce to recognize and respond to osteoarthritis must be a priority. This involves widespread training in the provision of evidence-based, culturally secure osteoarthritis care.
(2) Engage Indigenous voices in research efforts to better understand the experience of osteoarthritis from an Indigenous perspective. Through building the capabilities of Indigenous researchers to work in musculoskeletal health areas and adopting an Indigenous health lens, we can start to generate much-needed knowledge on the impact of osteoarthritis from the perspective of Indigenous communities.
(3) Improve access to culturally secure osteoarthritis care for Indigenous communities. Cultural security in health care occurs when services are offered in a way that will not compromise the cultural rights, values, beliefs, knowledge systems and expectations of Indigenous peoples [23,24]. Embedding these principles into the structures, policies and workforce of health services is essential to improve access to osteoarthritis care so that Indigenous peoples can remain active, healthy members of their communities [23,24].

It is now time to take musculoskeletal health off the backburner and recognise the central role that osteoarthritis and joint pain plays in managing chronic disease in Indigenous communities. We need to keep Indigenous peoples on their feet, so they can walk the path to improved health and wellbeing. 

## Figures and Tables

**Table 1:** Call to Action: Osteoarthritis and Indigenous Communities.

Why Is This Topic Important?	What Is the Issue?	How can We Fix the Issue?	Who Should Be Responsible?
(1) World-wide, osteoarthritis is a leading cause of activity restriction. (2) People with osteoarthritis live shorter lives with lower quality of life. (3) Indigenous populations within countries of similar socio-political context (Australia, Canada, New Zealand, USA) experience higher prevalence of osteoarthritis (up to 32%), greater burden of disease and access care at lower rates.	(1) Osteoarthritis is an under-recognised and under-researched area of Indigenous health. Osteoarthritis plays an important role in chronic disease management. (2) The impact of osteoarthritis and the related healthcare needs of Indigenous communities have not been investigated. Indigenous voices must be heard and supported in osteoarthritis research and service provision.	(1) To raise the osteoarthritis agenda in Indigenous health we must engage meaningfully and collaboratively with communities to: (i) Recognise the interrelated nature of osteoarthritis and chronic disease. (ii) Understand osteoarthritis through an Indigenous lens. (iii) Design and implement culturally secure osteoarthritis care. (iv) Build Indigenous capacity in the field.	(1) Funding bodies and policy makers need to prioritise musculoskeletal health research for Indigenous people as well as build capabilities for health services to provide culturally secure osteoarthritis care. (2) Researchers need to ensure that ethical principles of Indigenous health research are upheld throughout their research practices. This includes significant improvement in community.
